# Cross-sectional field study comparing hippocampal subfields in patients with post-traumatic stress disorder, major depressive disorder, post-traumatic stress disorder with comorbid major depressive disorder, and adjustment disorder using routine clinical data

**DOI:** 10.3389/fpsyg.2023.1123079

**Published:** 2023-06-13

**Authors:** Thiemo Knaust, Matthias B. D. Siebler, Dagmar Tarnogorski, Philipp Skiberowski, Helge Höllmer, Christian Moritz, Holger Schulz

**Affiliations:** ^1^Center for Mental Health, Bundeswehr Hospital Hamburg, Hamburg, Germany; ^2^Department of Radiology, Bundeswehr Hospital Hamburg, Hamburg, Germany; ^3^Department of Medical Psychology, University Medical Center Hamburg-Eppendorf, Hamburg, Germany

**Keywords:** hippocampal subfields, posttraumatic stress disorder (PTSD), depression, adjustment disorder, FreeSurfer, hippocampus, major depressive disorder (MDD)

## Abstract

**Background:**

The hippocampus is a central brain structure involved in stress processing. Previous studies have linked stress-related mental disorders, such as post-traumatic stress disorder (PTSD) and major depressive disorder (MDD), with changes in hippocampus volume. As PTSD and MDD have similar symptoms, clinical diagnosis relies solely on patients reporting their cognitive and emotional experiences, leading to an interest in utilizing imaging-based data to improve accuracy. Our field study aimed to determine whether there are hippocampal subfield volume differences between stress-related mental disorders (PTSD, MDD, adjustment disorders, and AdjD) using routine clinical data from a military hospital.

**Methods:**

Participants comprised soldiers (*N* = 185) with PTSD (*n* = 50), MDD (*n* = 70), PTSD with comorbid MDD (*n* = 38), and AdjD (*n* = 27). The hippocampus was segmented and volumetrized into subfields automatically using FreeSurfer. We used ANCOVA models with estimated total intracranial volume as a covariate to determine whether there were volume differences in the hippocampal subfields cornu ammonis 1 (CA1), cornu ammonis 2/3 (CA2/3), and dentate gyrus (DG) among patients with PTSD, MDD, PTSD with comorbid MDD, and AdjD. Furthermore, we added self-reported symptom duration and previous psychopharmacological and psychotherapy treatment as further covariates to examine whether there were associations with CA1, CA2/3, and DG.

**Results:**

No significant volume differences in hippocampal subfields between stress-related mental disorders were found. No significant associations were detected between symptom duration, psychopharmacological treatment, psychotherapy, and the hippocampal subfields.

**Conclusion:**

Hippocampal subfields may distinguish stress-related mental disorders; however, we did not observe any subfield differences. We provide several explanations for the non-results and thereby inform future field studies.

## 1. Introduction

The German Armed Forces, also known as the Bundeswehr, face regular instances of soldiers experiencing mental disorders following deployment. Post-traumatic stress disorder (PTSD) is one of the most common mission-related mental disorders, followed by Adjustment Disorder (AdjD), and Major Depressive Disorder (MDD; Kowalski et al., 2012). According to the 64th annual report of the Parliamentary Commissioner for the Armed Forces, the incidence of deployment-related mental illnesses among soldiers remains consistently high (German Bundestag, 2022). In 2022, out of 305 deployment-related mental illness cases, 197 were identified as PTSD. The average prevalence of PTSD among Bundeswehr soldiers 12 months after returning from deployment was 2.9% (Wittchen et al., 2012). In contrast, the prevalence among veterans of the U.S. military ranges from 2 to 15%, depending on the mission’s specifications (Na et al., 2023). The differences in prevalence rates are multifactorial, with one possible reason being the relatively longer deployment time and higher deployment burden experienced by U.S. soldiers compared to German troops.

The hippocampus (HC) is a crucial brain structure associated with the pathogenesis of PTSD, depression, and other stress-related disorders. As an important part of the limbic system, HC plays a critical role in consolidating and retrieving memories, regulating emotions, and learning processes (Franklin and Grossberg, 2017; Hainmueller and Bartos, 2020). The HC is hypothesized to play a key role in the neuroendocrine stress response in processing trauma-related stimuli. Several studies have indicated that PTSD [for reviews, see del Casale et al. (2022); Bromis et al. (2018)] and MDD [for a review, see Sun et al. (2023)] may lead to small HC volumes globally as well as specific morphological alterations in HC subfields (PTSD: Averill et al., 2017; Hayes et al., 2017; Chen et al., 2018; Postel et al., 2019, 2021; Zhang et al., 2021; MDD: Brown et al., 2019; Han et al., 2019; Roddy et al., 2019; Yao et al., 2020; Twait et al., 2022).

Stress-related mental disorders such as PTSD and MDD share similar symptoms (World Health Organization, 2018). Similar to many mental disorders, diagnosis depends solely on patients’ ability to describe their cognitive and emotional experiences (Koutsouleris et al., 2022). Therefore, there is a growing interest in utilizing imaging-based data to support a more accurate diagnosis in the long term (Serra-Blasco et al., 2021). In this clinical field study, we aimed to take a first step toward this goal by investigating whether there are significant differences in HC subfield volumes among patients with PTSD, MDD, PTSD with comorbid MDD, and AdjD using routinely acquired MRI data.

### 1.1. Hippocampal subfield alterations in post-traumatic stress disorder, depression, and adjustment disorder

#### 1.1.1. Structural imaging of the hippocampus and post-traumatic stress disorder

PTSD is a complex stress-related mental disorder that arises from a specific traumatic event, and its symptoms include intrusion, avoidance, hyperarousal, sleep and mood disturbances, and difficulty concentrating (World Health Organization, 2018). The HC is considered to play a central role in the pathophysiology of PTSD, and meta-analyses have provided evidence suggesting that PTSD is associated with a smaller hippocampal volume (O’Doherty et al., 2015; Bromis et al., 2018; Logue et al., 2018).

O’Doherty et al. (2015) reported significant reductions in HC volumes for patients with PTSD (*N* = 676) compared to non-traumatized controls (*N*_NTC_ = 460, *g* = 0.49), traumatized controls (*N*_TC_ = 487, *g* = 0.37), and the sub summation of non-traumatized and traumatized healthy controls (*g* = 0.42). However, these results should be interpreted cautiously because they may represent substantial heterogeneity (*I*^2^ = 42.7, *I*^2^ = 61.9, and *I*^2^ = 58.6, respectively). With more studies included and a larger sample size (*N*_PTSD_ = 2,689, *N*_NTC_ = 2,260, *N*_TC_ = 1,646), Bromis et al. (2018) found similar results (PTSD vs. NTC: *g* = 0.60, *I*^2^ = 0.53; PTSD vs. TC: *g* = 0.24, *I*^2^ = 0.55; PTSD vs. NTC + TC: *g* = 0.43, *I*^2^ = 0.58). One possible explanation for the heterogeneity could be the different populations (military vs. civilian), different image acquisition methodologies, different image processing methods (manual vs. automatic), and heterogeneous sample characteristics.

Logue et al. (2018) conducted a meta-analysis to address some of these shortcomings. They only included studies that used a standardized image analysis and quality control pipeline developed by the ENIGMA consortium. All included studies used FreeSurfer, a software-based automated volumetric program (Sämann et al., 2022). The authors further speculated that the results of previous meta-analyses may overestimate the true effect size due to the file drawer problem (Logue et al., 2018). Therefore, their consortium can contribute to existing knowledge by using unpublished data from multiple countries, which is likely to result in smaller but more unbiased effect sizes. Results showed a significant reduction in hippocampal volume in patients with PTSD (*N* = 780) compared to the sum of traumatized and non-traumatized controls (*N* = 1,062, *d* = 0.17; *I*^2^ = 0.00). They also conducted a subgroup analysis comparing military and civilian populations. The results showed a higher impact in civilians (*d* = 0.21, *p* = 0.003) than in military samples (*d* = 0.11, *p* = 0.110).

Recent studies have suggested examining hippocampal subfields in addition to the whole hippocampus (Weis et al., 2021). The subfields may provide a more nuanced understanding of the hippocampus’s functionality and afford greater precision to differentiate between patients with PTSD and traumatized and non-traumatized healthy controls (Chen et al., 2018; Postel et al., 2021; Zhang et al., 2021) and might even help differentiate between other mental disorders. Previous studies found a significantly smaller CA1 in patients with PTSD than in traumatized and non-traumatized controls (Chen et al., 2018; Postel et al., 2021), while other studies found a significantly smaller dentate gyrus (DG) compared to non-traumatized controls (Hayes et al., 2017; Postel et al., 2019; Zhang et al., 2021). Although less frequently reported, some studies have found negative correlations between the clinician-administered PTSD Scale and the hippocampus-amygdala transition area (HATA; Averill et al., 2017) and smaller volumes of CA4 compared to traumatized controls (Hayes et al., 2017). All studies used the FreeSurfer software. Nevertheless, the results should be interpreted with caution because of differences in the population (military: Averill et al., 2017; Hayes et al., 2017; Chen et al., 2018; civilian: Postel et al., 2019, 2021; Zhang et al., 2021), type of trauma, and sample sizes. However, they could be interpreted as preliminary evidence for particularly smaller CA1 and DG in patients with PTSD than in healthy controls.

Of note, the volume differences in the hippocampus and its subfields between PTSD patients and controls may not necessarily be a consequence or vulnerability factor for the development of PTSD. However, previous studies have shown conflicting results (Gilbertson et al., 2002; Kühn et al., 2021).

#### 1.1.2. Structural imaging of the hippocampus and major depressive disorder

MDD is the third most common mental illness among German soldiers and affects millions of people worldwide. In Germany, the lifetime prevalence of MDD ranges from 9.9% (Kessler et al., 2003) to 15% (Streit et al., 2022), while, in the United States, it is 20.6% (Hasin et al., 2018). Depressive episodes are characterized by a period of depressed mood or decreased interest in activities for most of the day, lasting at least 2 weeks, accompanied by other symptoms such as difficulty concentrating, feelings of worthlessness or excessive guilt, recurrent thoughts of death or suicide, changes in appetite or sleep, and more (World Health Organization, 2018).

The causes of MDD are multifactorial and complex; despite intensive research, the neurobiological effects on the brain and pathophysiological mechanisms of MDD are not yet fully understood (Yao et al., 2020). As part of the limbic system and the hypothalamic–pituitary–adrenal (HPA) axis, the HC plays a crucial role in the pathogenesis of MDD. In addition to its function in declarative memory processes, it is also involved in emotion regulation, motivational behavior, and the neuroendocrine stress response (Oyarce et al., 2020). Similar to the pathogenesis of PTSD, dysregulation of the HPA axis leads to increased cortisol release subsequently HC atrophy (Campbell and MacQueen, 2004).

Among other brain structures, changes in the HC are the most stable findings in brain volume research (Bromis et al., 2018; Oyarce et al., 2020), although, similar to PTSD, there is considerable heterogeneity. Bromis et al. (2018) reported significant reductions in hippocampal volume of patients with MDD (*N* = 1,377) compared to healthy controls (*N* = 1,281, *g* = 0.47, *I*^2^ = 0.67). Oyarce et al. (2020) reported similar results (*N*_MDD_ = 1737; *N*_Control_ = 2,142; mean volume difference = 0.20; *p* < 0.001; *I*^2^ = 0.86). The heterogeneity might be explained by methodological differences (image acquisition and image processing) and different clinical characteristics, such as severity, duration, onset-date, and recurrence of MDD (Bromis et al., 2018; Oyarce et al., 2020).

Some authors argue that examining the HC subfields may provide a more nuanced understanding to differentiate patients with MDD from healthy controls and to help distinguish it from other mental disorders (Sun et al., 2023). Here, a recent meta-analysis of HC subfields found significantly smaller left CA3 (included studies: *k* = 8, *I*^2^ = 0.71) and CA4 (included studies: *k* = 7, *I*^2^ = 0.69) and increased right HATA (included studies: *k* = 2, *I*^2^ = 0.27) in patients with MDD than in healthy controls. Furthermore, the authors conducted indirect volume comparisons between patients with schizophrenia and MDD by synthesizing studies that compared each group to healthy controls, but no significant differences were found (Sun et al., 2023). However, the heterogeneity, number of included studies, and methodology shortcoming, such as not controlling for intracranial volume (ICV), limit the interpretation of results. Nevertheless, the authors emphasize the need for head-to-head comparisons between patients with MDD and other mental disorders using a subfield-level examination of the HC to improve our understanding of the pathophysiology, which could lead to a more accurate diagnosis in the long term.

#### 1.1.3. Structural imaging of the hippocampus and post-traumatic stress disorder with comorbid major depressive disorder

PTSD and MDD are recognized as distinct mental disorders by the World Health Organization (2018), although they are often comorbid (Flory and Yehuda, 2015). While MDD as the primary diagnosis of comorbid PTSD is less common (1.4%; Dold et al., 2017), individuals with PTSD as the primary diagnosis also suffer from MDD. A meta-analysis of 57 studies (Rytwinski et al., 2013) showed that MDD co-occurred in 52% of PTSD cases. Furthermore, comorbid PTSD and MDD are significantly associated with increased distress and a more chronic course of impairment and are more prevalent among military personnel than the civilian population. Therefore, one might speculate that PTSD comorbid with MDD might be associated with more significant alterations than patients without PTSD or MDD comorbidities.

In this regard, the ENIGMA consortium conducted a study examining structural differences between individuals with comorbid PTSD and MDD (*N* = 621), PTSD-only (*N* = 384), MDD-only (*N* = 138), and controls without MDD or PTSD (*N* = 1,120; Salminen et al., 2019). The results indicated a significantly reduced volume of the hippocampal tail and CA1 in individuals with depression compared to controls. Additionally, the comorbid PTSD with depression group had a significantly reduced volume of CA1 not only compared to controls but also to the PTSD-only and MDD-only subgroups, especially in the military subsample. The authors concluded that comorbid PTSD and MDD might represent a unique biological phenotype with a particular vulnerability in the CA1 region. However, the results have been published as a preprint and should be interpreted cautiously until peer review is complete.

#### 1.1.4. Structural imaging of the hippocampus and adjustment disorder

According to ICD-11 criteria, AdjD is a maladaptive reaction to an identifiable psychosocial stressor or multiple stressors (e.g., divorce, illness, or conflicts at work) that usually emerges within a month of the stressor (World Health Organization, 2018). The disorder is characterized by preoccupation with the stressor, its consequences, or constant rumination about its implications. Patients cannot functionally adapt to stressors that cause significant impairment in everyday personal, family, and social life. The symptoms are not better explained by another mental disorder (e.g., MDD or PTSD) and typically resolve within 6 months unless the stressor persists longer (World Health Organization, 2018).

The prevalence in Germany is nationwide at about 1% (Maercker et al., 2012), and it is one of the most common diagnoses in the German Armed Forces after deployment (Kowalski et al., 2012).

We are unaware of any evidence available on the subfield-level examination of HC concerning AdjD. However, evidence suggests that AdjD is a stress-related mental disorder likely to be less severe (O’Donnell et al., 2016; Morgan et al., 2022). Therefore, it can be assumed that due to the potential lower severity of AdjD and the typically shorter duration of stress exposure, AdjD may be less affected by alterations in HC subfields than PTSD or MDD.

### 1.2. Anatomy of the hippocampus

HC can be macroscopically segmented into head, body, tail, and fissure (Sämann et al., 2022). Its main structures are the DG, cornu ammonis (CA), and subiculum (SUB). Histologically, the DG consists mainly of a granular cell layer (GC) surrounded by a molecular layer (ML). Other structures not part of HC are the presubiculum, parasubiculum, and entorhinal cortex. Furthermore, the main HC structures can be sub-segmented into subfields (Iglesias et al., 2015; Sämann et al., 2022).

HC is characterized by a higher-than-average concentration of glucocorticoid receptors, which makes it particularly sensitive to stress since elevated levels of glucocorticoids and excitatory neurotransmitters have a toxic effect on HC (McEwen et al., 1997). Coherent experiments on rodents have shown that the neurotoxic effect of stress can lead to the atrophy of HC (Bremner et al., 1995). Neurotoxicity can result in suppressed neurogenesis, decreased dendritic branching, and reduced synaptic or neuronal plasticity, which are mechanisms that can lead to a small HC volume (Wang et al., 2010). Nevertheless, a characteristic feature of HC is the plasticity of the organ (Franklin and Grossberg, 2017) by which HC neurons can recover from stress-induced atrophy. A previous study suggests that the neurotoxic effect of stress can be modulated or blocked not only with pharmacological interventions (Duman, 2004), and psychotherapy might lead to an increase in HC volume (Manthey et al., 2021).

Some HC subfields seem to be more effected by the neurotoxicity of stress than others, but neither PTSD (Chen et al., 2018; Postel et al., 2021; Zhang et al., 2021) nor MDD (Sun et al., 2023) nor AdjD can finally be attributed to specific HC subfields yet (Salminen et al., 2019).

### 1.3. Research questions

Previous studies have focused on the total HC volume and found smaller volumes in patients with PTSD and MDD than in healthy controls (O’Doherty et al., 2015; Bromis et al., 2018; Logue et al., 2018; Oyarce et al., 2020). However, interpreting these findings requires consideration of substantial heterogeneity. Moreover, it remains unclear how these results can be translated to the clinical inpatient routine, where healthy controls are typically unavailable. In this context, a more nuanced understanding of the potential differences between stress-related mental disorders would greatly benefit supporting accurate long-term diagnosis (Weis et al., 2021; Sun et al., 2023). Examining HC subfield volumes could be a promising tool for achieving this goal.

Previous studies have found that patients with PTSD have smaller CA1 and DG regions than healthy controls (Hayes et al., 2017; Chen et al., 2018; Postel et al., 2019, 2021; Zhang et al., 2021). Although some evidence for smaller CA2/3 and HATA regions exists in patients with PTSD, these findings have been less frequently reported (Averill et al., 2017; Hayes et al., 2017). A recent meta-analysis by Sun et al. (2023) revealed initial evidence for a smaller CA2/3, CA4, and increased HATA region in patients with MDD compared to healthy controls. Furthermore, their results showed no significant differences in HC subfield volumes between patients with MDD and schizophrenia. However, this null result should be interpreted cautiously, as they conducted indirect comparisons since they identified no study that directly compared patients with MDD and schizophrenia. Accordingly, the authors encourage future research to compare different mental disorders directly.

In light of these findings, we conducted a field study to investigate whether stress-related mental disorders (PTSD, MDD, PTSD+MDD, and AdjD) differ significantly in HC subfield volumes (CA1, CA2/3, and DG) when controlling for estimated intracranial volume (eTIV) using MRI scans from routine clinical data. We included AdjD in our study design, as they are highly prevalent in military populations. Moreover, it can be assumed that patients with AdjD, who are likely exposed to stress for a shorter duration and with lesser severity, may be less affected by HC subfield alterations than individuals with PTSD or MDD.

Our exploratory analyses examined whether self-reported symptom duration, medication treatment, and psychotherapeutic experience are associated with CA1, CA2/3, and DG volumes and whether they may influence potential differences between stress-related mental disorders.

## 2. Materials and methods

The local ethics committee of the Chamber of Physicians in Hamburg, Germany (Ref. No.: PV7098), the Administrative Data Protection Officer of the Bundeswehr Hospital Hamburg, and the members of the research conference of the Bundeswehr Medical Academy Munich, Germany (44 K2-S-322224) approved the study design.

### 2.1. Sample and clinical measures

The following inclusion criteria were defined for this retrospective cross-sectional cohort study: patients had to meet one of the following ICD-10 diagnoses, as assessed by a specialist in psychiatry and a licensed psychotherapist: a single episode of major depressive episode in mild (F32.0)/moderate (F32.1)/severe (F32.2) severity, a recurrent major depressive episode in mild (F33.0)/moderate (F33.1)/severe (F33.2) severity, PTSD (F43.1), or AdjD (F43.2). We used psychiatrically indicated cMRI scans obtained during the inpatient stay (to exclude somatic brain changes initially) and evaluated them using FreeSurfer 6.0 to get volumes of the hippocampal subfields. Exclusion criteria included previous intracranial injury, CNS disorders such as epilepsy, multiple sclerosis, intracranial tumors, psychosis, and alcohol or drug dependence.

The sample recruited from the clinical routine comprised 185 patients (162 men, 23 women) who received inpatient psychiatric treatment at the Bundeswehr Hospital Hamburg from January 2014 to March 2019 and underwent a cMRI scan during the examination period, which was performed using the same MRI scanner (see section 2.2). Subjects were divided into the following four patient groups for subsequent hypothesis testing: (i) patients with diagnosed MDD (*n* = 70, 37.8%), (ii) patients with diagnosed PTSD (*n* = 50, 27.0%), (iii) patients with diagnosed PTSD and comorbid MDD (*n* = 38, 20.6%), and (iv) patients with diagnosed adjustment disorder (*n* = 27, 14.6%). The patients’ ages ranged from 17 to 61 years (*M* = 31.96, SD = 8.96). Of the sample, 69 patients (37.3%) were treated with psychopharmacological medication, and 85 patients (45.9%) had undergone psychotherapy (including both outpatient and inpatient psychotherapy), with general dichotomous extraction categories formed for data protection reasons. The reported symptom duration was extracted (*M* = 42.68 months, SD = 48.1, range: 1–269 months), although the duration of symptoms highly depends on the specific mental disorder. However, previous empirical studies have suggested an association between symptom duration and hippocampal volume alterations (Bromis et al., 2018; Oyarce et al., 2020). Additional sociodemographic variables, such as gender, education, military rank, and previous psychotherapeutic experience, are also recorded and reported in Table 1.

**Table 1:** Sociodemographic variables of the sample.

	PTSD (*N* = 50)		MDD (*N* = 70)		PTSD + MDD (*N* = 38)		AdjD (*N* = 27)			
Variable	*n*	%	*n*	%	*n*	%	*n*	%	*χ*^2^ (d*f*)	*p*
Sex									1.68(3)	0.640
Male	44	88.0	63	90.0	31	81.6	24	88.9		
Female	6	12.0	7	10.0	7	18.4	3	11.1		
Education									6.30(6)	0.390
University	9	18.8	12	17.4	2	5.3	5	18.5		
Highschool	6	12.5	15	21.7	8	21.1	7	25.9		
Middle school	33	68.8	42	60.9	28	73.7	15	55.6		
Military rank									7.69(6)	0.261
OF	10	20.0	15	21.4	4	10.5	7	25.9		
OR 5–9	22	44.0	32	45.7	26	68.4	11	40.7		
OR 1–4	18	36.0	23	32.9	8	21.1	9	33.3		
Prev. Psycho									22.8 (3)	<0.001
Yes	28	56.0	25	35.7	27	71.1	5	18.5		
No	22	44.0	45	64.3	11	28.9	22	81.5		

OF, officers; OR 5–9, noncommissioned officers; OR 1–4, enlisted ranks; Prev. Psycho, previous psychotherapy experience.

The medical and psychiatric histories of the participants were obtained from their medical reports at the Center for Mental Health of the Bundeswehr Hospital Hamburg. According to the data security concept, access to these reports was restricted to authorized personnel. MRI data and MRI reports were obtained from the Radiology department of Bundeswehr Hospital Hamburg. The psychiatric diagnoses were assigned by a specialist in psychiatry and a licensed psychotherapist based on the ICD-10 criteria, which were assessed through anamnesis interviews and reevaluated during treatment. Psychometric test procedures were frequently used to support the clinically assigned diagnoses but were not obligatory. Unfortunately, due to data protection regulations, we did not have access to the results of the psychometric tests (see 4.1, limitations section). A radiology specialist assessed MRI data.

### 2.2. Analysis and processing of cMRI data

Magnetic resonance imaging (MRI) scans were acquired using a 3 T Siemens Skyra MRI scanner (Siemens AG Medical Solutions, Erlangen, Germany) at the Radiology Department of the Bundeswehr Hospital, using a 20-channel head–neck coil. Along with other MRI sequences, a sagittal three-dimensional gradient-echo T1-weighted sequence called magnetization-prepared rapid gradient echo (MPRAGE) was obtained for structural analysis. The parameters of the MPRAGE sequence were as follows: TR of 2300 ms, TE of 2.3 ms, matrix size of 256 × 256, voxel size of 0.9 mm^3^, and 192 slices in the sagittal plane.

The reconstruction of images and automated delineation of the whole HC and its surrounding associated structures into subfields were performed using version 6.0 of the FreeSurfer software.^1^ This software utilizes a Bayesian modeling approach that predicts the location of neuroanatomical labels based on probabilistic atlases and learns the locations of manual hippocampal segmentations from training subjects (Iglesias et al., 2015). The automated segmentations were validated against manual morphometric measurements of ultra-high-resolution scans and demonstrated improved interstudy comparability (Hayes et al., 2017; Sämann et al., 2022).

FreeSurfer 6.0 segmented the left and right HC into different subfields: for the HEAD, these were parasubiculum, presubiculum-head, subiculum-head, CA1-head, CA2/3-head, CA4-head, GC-ML-DG-head, molecular_layer-HP-head, and hippocampus-amygdala transition area (HATA); for the BODY, the subfields were presubiculum-body, subiculum-body, CA1-body, CA2/3-body, CA4-body, GC-ML-DG-body, molecular_layer-HP-body, and fimbria; for the TAIL, the subfields were hippocampal tail and for the FISSURE, hippocampal-fissure. Owing to a lack of distinguishing contrast and the small size of the CA2 subfield, CA2, and CA3 were combined and discussed as CA 2/3. Subfields were combined to form larger subfield structures, including subiculum, presubiculum, parasubiculum, CA1, CA2/3, CA4, and GC-ML-DG (including the DG and molecular layer), respectively, to ensure precise volume quantification. HATA, fimbria, HC tail, and HC fissure were also included.

Individual differences in brain and HC volumes are influenced by sex, age, and head size (Nerland et al., 2022). Therefore, adjusting for intracranial volume (ICV) to compare HC subfield volumes between individuals is essential. FreeSurfer software was used to calculate the estimated total intracranial volume (eTIV), which is equivalent to manually generated ICV (Buckner et al., 2004) and was included as a covariate in our analysis (O'Brien et al., 2011).

### 2.3. Statistical analysis

All statistical calculations were conducted using IBM SPSS Statistics for Windows version 28 (IBM Corp. Released 2021, Armonk, N.Y., USA). Based on previous studies, we selected the following hippocampal subfields as variables of interest: (i) CA1, (ii) CA2/3, and (iii) DG. As previous studies did not conclusively provide information on a specific hemisphere, we defined the total volume of the CA1, CA2/3, and DG subfields as the primary outcome variable by adding the volumes of the left and right hemispheres. Inferential statistics were only conducted for CA1, CA2/3, and DG to reduce the likelihood of false positives due to multiple dependent variables.

For each dependent variable (CA1, CA2/3, and DG), we conducted a one-factorial analysis of covariance (ANCOVA), with the patient groups (MDD, PTSD, PTSD+MDD, and AdjD) as the independent variable and eTIV as a covariate. If a significant main effect for the factor patient groups was found, we conducted *a-priori* planned contrast analyses to examine whether the volume of each dependent variable was significantly smaller in patients with (i) MDD, (ii) PTSD, and (iii) PTSD+MDD than AdjD.

**Figure 1:**
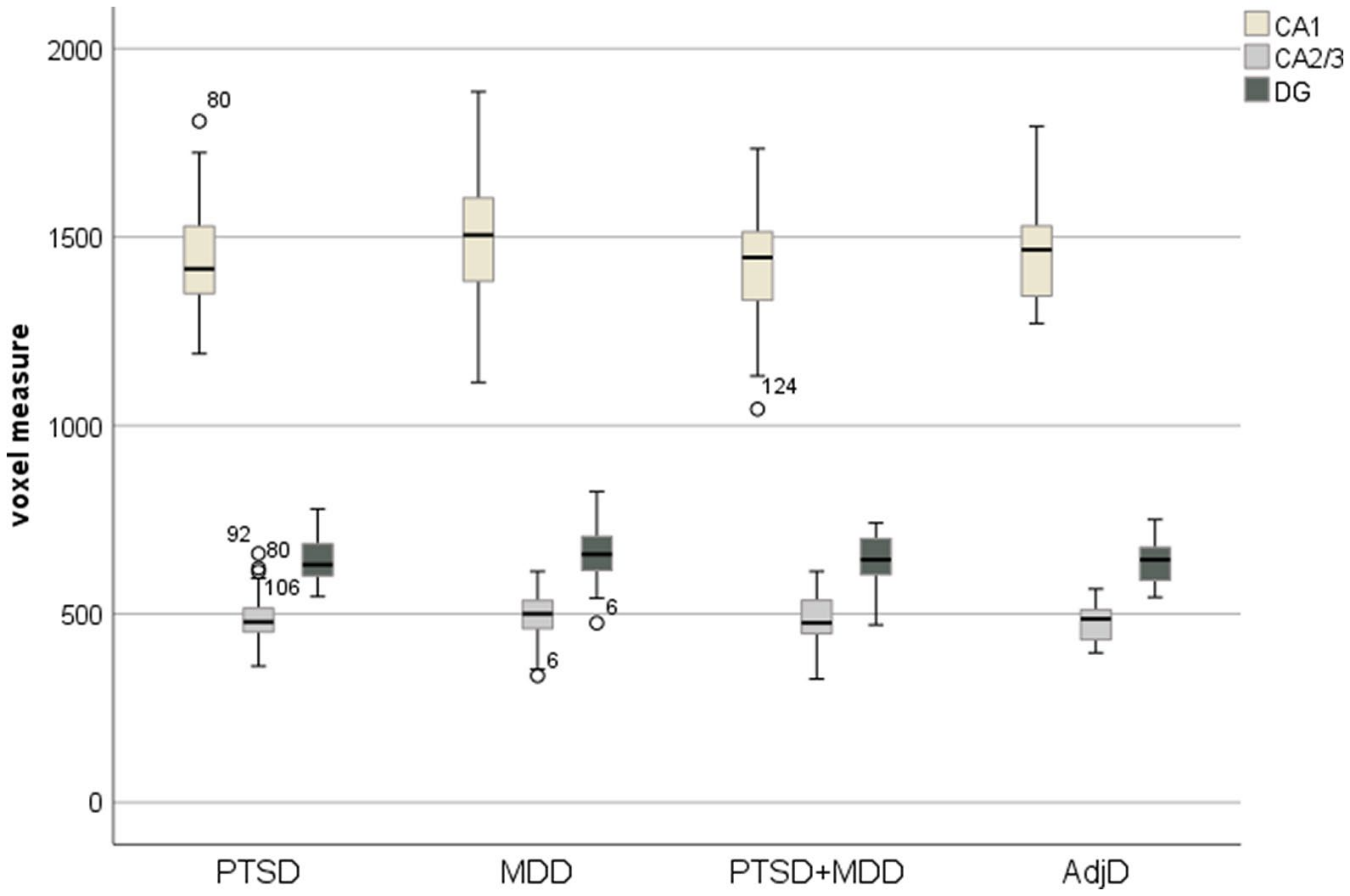
Boxplot with CA1, CA2/3, and DG separated by factor patient group.

We used histograms separated by the patient group to test the normal distribution assumption. Visual inspection did not show a clear normal distribution in all cases. Therefore, we repeated the analysis using the bootstrapping procedure (bootstrap samples: *k* = 1,000 with bias-corrected confidence intervals; Field, 2018) and reported the bootstrapped parameter estimates in Supplementary Table S1. No substantial differences were observed. We used boxplots for each outcome variable separated by the patient group to perform an outlier analysis (see Figure 1). If outliers were identified, we conducted covariance analysis with and without outliers (Pollet and Van Der Meij, 2017). However, the results obtained with and without the outliers did not differ significantly. Therefore, we only reported the results with outliers in the manuscript, whereas Supplementary Table S2 reports the results with and without outliers.

Although our inferential analyses focused on CA1, CA2/3, and DG, we computed descriptive statistics for all hippocampal subfields to provide a more comprehensive overview of the data. Descriptive statistics, including means, standard deviations, and 95% confidence intervals, are reported in Supplementary Table S3. In addition to these statistics, we calculated eTIV-corrected effect sizes (using Cohen’s d for unequal-sized samples; Ellis, 2010) between the different stress-related mental disorders for each hippocampal subfield, separated by hemisphere, and presented them in Table 2.

**Table 2:** The eTIV corrected between-subjects effect sizes of the individual hippocampal subfields.

Variable	MDD vs. PTSD		MDD vs. PTSD+MDD		MDD vs. AdjD		PTSD vs. PTSD+MDD		PTSD vs. AdjD		PTSD+MDD vs. AdjD	
	MDiff	Cohen’s *d*	MDiff	Cohen’s *d*	MDiff	Cohen’s *d*	MDiff	Cohen’s *d*	MDiff	Cohen’s *d*	MDiff	Cohen’s *d*
Left												
Para	1.706	−0.157	−2.490	0.228	−0.031	0.003	−4.197	0.388	−1.737	0.161	2.459	−0.227
Pre	0.303	−0.008	−3.287	0.088	3.876	−0.104	−3.589	0.097	3.574	−0.097	7.163	−0.193
Subiculum	−4.131	0.091	−6.334	0.139	1.553	−0.034	−2.202	0.049	5.684	−0.126	7.886	−0.174
CA1	4.830	−0.070	16.830	−0.244	9.696	−0.141	12.000	−0.176	4.866	−0.072	−7.133	0.104
CA2/3	−1.812	0.068	−3.373	0.127	5.855	−0.220	−1.561	0.059	7.667	−0.291	9.228	−0.348
CA4	0.356	−0.013	−3.344	0.125	2.484	−0.093	−3.700	0.140	2.128	−0.081	5.828	−0.220
GC/DG	0.887	−0.035	−3.082	0.122	5.686	−0.225	−3.969	0.158	4.799	−0.192	8.768	−0.348
ML	−1.682	0.035	1.073	−0.022	5.462	−0.114	2.755	−0.058	7.144	−0.150	4.389	−0.092
HATA	1.100	−0.139	0.382	−0.048	1.626	−0.205	−0.719	0.091	0.526	−0.067	1.244	−0.158
Fimbria	−0.009	0.001	−3.299	0.182	6.394	−0.353	−3.308	0.183	6.385	−0.356	9.693	−0.536
HT	−7.329	0.117	1.615	−0.026	−16.262	0.260	8.945	−0.144	−8.933	0.144	−17.877	0.286
HF	−3.789	0.176	1.483	−0.068	−1.553	0.072	5.272	−0.245	2.236	−0.105	−3.036	0.141
Right												
Para	2.706	−0.288	0.077	−0.008	−0.939	0.100	−2.629	0.281	−3.646	0.391	−1.017	0.108
Pre	3.952	−0.126	4.866	−0.154	7.931	−0.252	0.915	−0.029	3.979	−0.127	3.064	−0.097
Subiculum	4.132	−0.105	−3.12	0.079	10.701	−0.270	−7.252	0.184	6.569	−0.167	13.821	−0.350
CA1	9.052	−0.127	10.251	−0.143	8.144	−0.114	1.199	−0.017	−0.908	0.013	−2.107	0.030
CA2/3	−0.566	0.019	−5.409	0.178	6.789	−0.225	−4.844	0.161	7.355	−0.246	12.199	−0.405
CA4	1.059	−0.041	−4.196	0.160	5.304	−0.203	−5.254	0.202	4.245	−0.164	9.500	−0.364
GC/DG	1.201	−0.041	−4.777	0.163	6.295	−0.215	−5.977	0.205	5.094	−0.176	11.071	−0.380
ML	2.825	−0.056	−0.956	0.019	9.161	−0.182	−3.781	0.075	6.336	−0.127	10.117	−0.201
HATA	1.703	−0.196	1.181	−0.135	3.511	−0.404	−0.522	0.060	1.808	−0.210	2.330	−0.269
Fimbria	1.846	−0.099	−0.267	0.014	2.351	−0.126	−2.113	0.114	0.505	−0.027	2.618	−0.141
HT	−0.641	0.010	−7.643	0.119	−3.762	0.059	−7.001	0.110	−3.121	0.049	3.880	−0.061
HF	1.010	−0.042	0.518	−0.022	10.161	−0.427	−0.491	0.021	9.151	−0.388	9.642	−0.625
Total (Left + Right)												
Para	4.413	0.256	2.413	0.139	0.970	0.056	6.826	0.397	5.383	0.315	1.443	0.084
Pre	4.254	0.102	1.579	0.040	11.807	0.333	2.675	0.043	7.552	0.123	10.227	0.165
Subiculum	0.001	0.001	9.453	0.120	12.254	0.156	9.454	0.121	12.253	0.157	21.707	0.276
CA1	13.882	0.109	27.081	0.211	17.840	0.140	13.199	0.104	3.958	0.031	9.241	0.072
CA2/3	2.378	0.047	8.782	0.171	12.644	0.247	6.405	0.126	15.022	0.296	21.427	0.419
CA4	1.415	0.032	7.540	0.168	7.788	0.174	8.954	0.201	6.373	0.144	15.327	0.343
GC/DG	2.088	0.042	7.859	0.156	11.981	0.238	9.946	0.199	9.893	0.199	19.840	0.396
ML	1.143	0.013	0.117	0.001	14.623	0.161	1.026	0.011	13.480	0.150	14.506	0.160
HATA	2.804	0.194	1.563	0.108	5.137	0.354	1.241	0.085	2.334	0.161	3.574	0.247
Fimbria	1.855	0.056	3.566	0.108	8.745	0.265	5.421	0.165	6.890	0.211	12.311	0.375
HT	7.971	0.066	6.027	0.050	20.025	0.166	1.943	0.016	12.054	0.101	13.997	0.116
HF	2.779	0.069	2.002	0.049	8.608	0.213	4.781	0.119	11.387	0.284	6.606	0.164

MDD, major depressive disorder; PTSD, post-traumatic stress disorder; PTSD + MDD, post-traumatic stress disorder with comorbid major depressive disorder; AdjD, adjustment disorder; Para, parasubiculum; Pre, presubiculum; CA, cornu ammonis; GC/DG, granular cell layer/dentate gyrus; ML, molecular layer; HATA, hippocampus-amygdala transition area; HT, hippocampal tail; HF, hippocampal fimbria.

### 2.4. Power analysis

A post-hoc power analysis was conducted using an adjusted alpha level of 0.017 and power of 80%, with *N* = 185 participants, one covariate (eTIV), and one independent variable (patient groups: MDD, PTSD, PTSD + MDD, and AdjD) for analysis of covariance. The results indicated that we could detect differences if the effect size were *f* ≥ 0.27 (0.10 = small effect size, 0.25 = medium effect size, 0.40 = large effect size; Cohen, 2013). Power analysis was performed using G*Power software (Faul et al., 2007). Given that we conducted three ANCOVAs, we applied the Bonferroni correction and adjusted the alpha level to *α* = 0.017.

## 3. Results

The ANCOVA results for the CA1, CA2/3, and DG subfields are presented in sections 3.1–3.3, respectively. For an overview of the results, see Table 3.

**Table 3:** Overview of the ANCOVA results with outliers.

Variable	Test statistics			
	*F*	(d*f*)	*p*	*η*_p_²
CA1				
eTIV	77.6	(1, 180)	<0.001	0.301
patient group	0.39	(3, 180)	0.301	0.006
symptom duration	0.87	(1, 143)	0.352	0.006
symptom duration × patient group	0.88	(3, 143)	0.453	0.018
medication	0.47	(1, 143)	0.494	0.003
medication × patient group	1.08	(3, 143)	0.356	0.022
prev psychotherapy	0.17	(1, 143)	0.681	0.001
prev psychotherapy × patient group	0.51	(3, 143)	0.670	0.011
CA2/3				
eTIV	59.7	(1, 180)	<0.001	0.249
patient group	0.96	(3, 180)	0.412	0.016
symptom duration	0.97	(1, 143)	0.325	0.007
symptom duration × patient group	0.21	(3, 143)	0.884	0.005
medication	0.19	(1, 143)	0.659	0.001
medication × patient group	0.73	(3, 143)	0.531	0.015
prev psychotherapy	0.09	(1, 143)	0.754	0.001
prev psychotherapy × patient group	0.92	(3, 143)	0.432	0.019
DG				
eTIV	98.6	(1, 180)	<0.001	0.354
patient group	0.85	(3, 180)	0.467	0.014
symptom duration	1.83	(1, 143)	0.178	0.013
symptom duration × patient group	0.68	(3, 143)	0.560	0.014
medication	0.69	(1, 143)	0.793	<0.001
medication × patient group	1.11	(3, 143)	0.347	0.023
prev psychotherapy	0.01	(1, 143)	0.910	<0.001
prev psychotherapy × patient group	0.79	(3, 143)	0.497	0.016

### 3.1. Cornu ammonis 1 subfield

One-factorial ANCOVA revealed a significant main effect for the covariate eTIV, *F*(1,180) = 77.6, *p* < 0.001, *η_p_*^2^ = 0.301, but no significant differences for the factor patient group, *F*(3,180) = 0.39, *p* = 0.301, *η_p_*^2^ = 0.006.

We added potential covariates (symptom duration, medication, and prior psychotherapeutic experience) to the ANCOVA models for the exploratory questions. We examined their effects and the interaction effects with the patient group factor on CA1. However, we found no significant associations: (i) symptom duration, *F*(1,143) = 0.87, *p* = 0.352, *η_p_*^2^ = 0.006, (ii) interaction of symptom duration × patient group, *F*(3,143) = 0.88, *p* = 0.453, *η_p_*^2^ = 0.018, (iii) medication, *F*(1,143) = 0.47, *p* = 0.494, *η_p_*^2^ = 0.003, (iv) interaction of medication × patient group, *F*(3,143) = 1.08, *p* = 0.356, *η_p_*^2^ = 0.022, (v) previous psychotherapeutic experience, *F*(1,143) = 0.17, *p* = 0.681, *η_p_*^2^ = 0.001, and (vi) interaction of previous psychotherapeutic experience × patient group, *F*(3,143) = 0.51, *p* = 0.670, *η_p_*^2^ = 0.011.

### 3.2. Cornu ammonis 2/3 subfield

The results of the one-factorial ANCOVA showed a significant effect for the covariate eTIV, *F*(1,180) = 59.7, *p* < 0.001, *η_p_*^2^ = 0.249, but no significant differences for the factor patient group, *F*(3,180) = 0.96, *p* = 0.412, *η_p_*^2^ = 0.016.

To address these exploratory questions, we applied the same procedure as that for subfield CA1. Again, we found no significant associations between these covariates and the volume of CA2/3: (i) symptom duration, *F*(1,143) = 0.97, *p* = 0.325, *η_p_*^2^ = 0.007, (ii) the interaction of symptom duration × patient group, *F*(3,143) = 0.21, *p* = 0.884, *η_p_*^2^ = 0.005, (iii) medication, *F*(1,143) = 0.19, *p* = 0.659, *η_p_*^2^ = 0.001, (iv) the interaction of medication × patient group, *F*(3,143) = 0.73, *p* = 0.531, *η_p_*^2^ = 0.015, (v) previous psychotherapeutic experience, *F*(1,143) = 0.09, *p* = 0.754, *η_p_*^2^ = 0.001, and (vi) the interaction of previous psychotherapeutic experience × patient group, *F*(3,143) = 0.92, *p* = 0.432, *η_p_*^2^ = 0.019.

### 3.3. Dentate gyrus subfield

The results of the one-factorial ANCOVA showed a significant effect for the covariate eTIV, *F*(1,180) = 98.6, *p* < 0.001, *η_p_*^2^ = 0.354, but no significant differences for the factor patient group, *F*(3,180) = 0.85, *p* = 0.467, *η_p_*^2^ = 0.014.

Regarding the exploratory questions, we found no significant associations between the covariates and the volume of DG: (i) symptom duration, *F*(1,143) = 1.83, *p* = 0.178, *η_p_*^2^ = 0.013, (ii) the interaction of symptom duration x patient group, *F*(3,143) = 0.68, *p* = 0.560, *η_p_*^2^ = 0.014, (iii) medication, *F*(1,143) = 0.06, *p* = 0.793, *η_p_*^2^ < 0.001, (iv) the interaction of medication × patient group, *F*(3,143) = 1.11, *p* = 0.347, *η_p_*^2^ = 0.023, (v) previous psychotherapeutic experience, *F*(1,143) = 0.01, *p* = 0.910, *η_p_*^2^ < 0.001, and (vi) the interaction of previous psychotherapeutic experience × patient group, *F*(3,143) = 0.79, *p* = 0.497, *η_p_*^2^ = 0.016.

## 4. Discussion

We found no significant differences in stress-related mental disorders between the CA1, CA2/3, and DG. One possible interpretation of these findings is that examining HC subfields using routine clinical data cannot effectively distinguish between PTSD, MDD, PTSD with comorbid MDD, and AdjD. This may be due to the difficulty in generalizing previous research to clinical practice or the limitations of our study design.

Despite the substantial heterogeneity, it is generally accepted that the total HC volume in patients with PTSD and MDD is smaller than that in healthy controls (O’Doherty et al., 2015; Bromis et al., 2018; Logue et al., 2018; Oyarce et al., 2020). A biopsychological explanation is that HC has a higher-than-average concentration of glucocorticoid receptors, making it particularly vulnerable to stress (McEwen et al., 1997). Studies in rodents have demonstrated that stress-induced neurotoxicity can result in HC atrophy (Bremner et al., 1995). However, it is unclear whether this is a stress-associated phenomenon in general or whether it differs between stress-related mental disorders. The latter would be particularly relevant in clinical settings where healthy controls are typically unavailable. Bromis et al. (2018) found no significant differences in total HC between patients with PTSD and MDD. Their results are limited because they conducted an indirect comparison and identified no studies that performed a head-to-head comparison.

Some authors have argued that examining HC subfields can provide a more nuanced understanding of hippocampal functionality, which may be differently affected by different mental disorders (Chen et al., 2018; Postel et al., 2021; Zhang et al., 2021; Sun et al., 2023). Although these studies found initial evidence for specific subfields (CA1, CA2/3, and DG), they also used healthy controls as a comparison group. Therefore, whether examining HC subfields could help differentiate stress-related mental disorders remains unanswered.

Our non-significant results indicate that smaller HC volumes (in total or at the subfield level) may represent a general stress response. Following this assumption, if any differences exist between stress-related mental disorders, the expected effect sizes are relatively small. Therefore, our study may be underpowered, although our sample was relatively large compared to previous studies (Averill et al., 2017; Ahmed-Leitao et al., 2019; Postel et al., 2019, 2021). Nevertheless, we included AdjD in our study design, assuming that due to the typically shorter duration and lower severity of stress exposure, AdjD might be less affected by alterations in HC subfields than PTSD and MDD. Contrary to our expectations, we did not find any significant differences. This may be interpreted as an additional indication that smaller HC subfields are associated with a more general stress response. However, this interpretation remains speculative because we did not include healthy controls.

Previous meta-analyses have revealed substantial heterogeneity and discussed whether this might be caused by variations in patient characteristics, such as symptom duration, drug treatment, and previous psychotherapy experience (Bromis et al., 2018; Sun et al., 2023). These covariates may influence the neuronal plasticity of HC subfields and impact alterations. Therefore, we implemented these variables as covariates in our ANCOVA models.

For example, prolonged symptom duration may lead to a chronic stress response, which has been associated with reduced hippocampal volume (Bremner et al., 1995; Duman, 2004). However, some studies failed to find associations between symptom duration and subfield volumes in patients with MDD (Cao et al., 2017). Our exploratory analysis revealed no significant associations. To examine these divergences, future longitudinal studies should examine the dose–response curve between symptom duration and hippocampal subfield alterations.

Similarly, psychopharmacological treatments, such as antidepressants or antipsychotics, have modulated hippocampal function and structure (Vermetten et al., 2003; Duman, 2004; Yao et al., 2020). However, our exploratory analysis again showed no significant correlation with the current psychopharmacological treatment. Differences in study design may explain this. For example, Vermetten et al. (2003) treated a therapy-naive sample exclusively with paroxetine for 36–48 weeks and allowed no other interventions. Therefore, our dichotomous operationalization may not be comprehensive enough to detect potential associations.

Finally, psychotherapy has been suggested to induce plastic changes in HC, potentially impacting its subfields. However, the empirical evidence supporting this assumption is mixed. Manthey et al. (2021) identified only one study in their systematic review that showed increased hippocampal volume following trauma-focused therapy. The authors concluded that the findings were too heterogeneous and scarce to allow for robust conclusions regarding the psychotherapeutic effect on HC morphology. Our exploratory analyses also found no significant associations between previous psychotherapeutic experience and CA1, CA2/3, or DG. Therefore, future intervention studies should empirically focus on the psychotherapeutic effect on hippocampal subfields.

### 4.1. Limitations

Our findings must be considered in the context of several limitations. The primary limitation was the absence of a healthy control condition. We focused on identifying differences between stress-related mental disorders with the long-term aim of improving diagnostic accuracy using routine clinical data. Nonetheless, we cannot conclusively determine whether we have identified smaller hippocampal volumes without a healthy control group for comparison. Typically, multiple healthy control conditions (trauma-exposed, trauma-unexposed) are necessary to conclude the cause and effect of exposure in cross-sectional samples (Gosnell et al., 2020; Siehl et al., 2020). We did not include a healthy control group, as they are not typically available in clinical settings. However, the interpretation of these results is limited, and future studies should replicate our results with multiple healthy controls.

Another limitation is that self-reported severity was not collected as a standard measure. Although some authors have found significant correlations between severity and HC subfields (Averill et al., 2017; Hayes et al., 2017), we were unable to empirically verify this relationship, which also limits the interpretation of the results. Additionally, the dichotomized assessment of previous psychotherapies and medication treatment may not have been sufficiently comprehensive to detect an association, limiting the significance of these parameters. Future studies should examine whether a more detailed assessment (e.g., number of outpatient therapy sessions, duration, and hospital stays) could affect hippocampal volume. In addition, we did not document the type of trauma experienced by the participants. While it is likely that most PTSD patients in our sample had a military-related trauma, because we only included soldiers, we could not empirically confirm this hypothesis. Because the type of trauma could potentially influence changes in hippocampal subfields, future studies should systematically examine the type of trauma experienced by the participants.

Finally, it should be noted that we had significantly fewer women than men in our sample. Although the ratio in our study is generally comparable to military samples, this may also have influenced the results.

## 5. Conclusion

In conclusion, our study demonstrates the potential of using routine clinical data to investigate differences in HC subfield volumes in patients with PTSD, MDD, and AdjD, with the goal of improving the diagnosis of stress-related mental disorders. Current diagnosis relies heavily on patient report, and the incorporation of neuroimaging data could significantly enhance our understanding of these disorders. Despite previous studies suggesting that AdjD may have lower symptom severity and stress exposure, our findings did not reveal significant volume differences between stress-related mental disorders.

Despite the limitations of the present study, our findings raise questions about the applicability of previous research results to clinical practice without healthy controls. To address these issues, future larger multicenter studies should include routine inpatient MRI scans and multiple healthy controls into their study design. Such studies are crucial to advancing our understanding of stress-related mental disorders and improving their diagnosis and long-term treatment.

## Data availability statement

The raw data that supports the conclusions of this article are not publicly available due to data policy reasons. However, the data can be made available upon reasonable request to the corresponding author.

## Ethics statement

The studies involving human participants were reviewed and approved by local ethics committee of the chamber of physicians, Hamburg, Germany (Ref. no.: PV7098). Written informed consent for participation was not required for this study in accordance with the national legislation and the institutional requirements.

## Author contributions

TK contributed to the study design and performed the statistical analysis. MS contributed to the study design, performed scientific research, and carried out software-based volumetric analysis of the MRI-data. TK and MS took the lead in drafting the manuscript. DT contributed to the study design, carried out the scientific research, and contributed to the manuscript. PS participated in the data processing and software-based volumetric analysis of the MRI-data. HH supervised the psychiatric diagnostic process and provided the psychiatric reports. CM supervised the radiologic diagnostic process and provided the radiologic reports. HS conceived the study, and participated in its design and coordination and helped to draft the manuscript. All authors provided critical feedback and helped shape the research, analysis, and manuscript.

## Funding

This study was funded by the German Ministry of Defense; Bundeswehr Medical Academy Munich, Germany (44 K2-S-32 2224).

## Conflict of interest

The authors declare that the research was conducted in the absence of any commercial or financial relationships that could be construed as a potential conflict of interest.

## Publisher’s note

All claims expressed in this article are solely those of the authors and do not necessarily represent those of their affiliated organizations, or those of the publisher, the editors and the reviewers. Any product that may be evaluated in this article, or claim that may be made by its manufacturer, is not guaranteed or endorsed by the publisher.

## Footnotes

^1^http://surfer.nmr.mgh.harvard.edu

## Glossary

AdjD: Adjustment Disorder
ANCOVA: Analysis of Covariance
CA: Cornu Ammonis; Ammon’s Horn
CAPS: Clinician-Administered PTSD Scale
DG: Dentate Gyrus
eTIV: estimated Total Intracranial Volume
GC: Granular Cell Layer
HATA: Hippocampus-Amygdala Transition Area
HC: Hippocampus
HF: Hippocampal Fimbria
HT: Hippocampal Tail
ICD: International Classification of Diseases
ICV: Intracranial Volume
M: Mean
MDD: Major Depressive Disorder
ML: Molecular Layer
MPRAGE: Magnetization Prepared Rapid Gradient Echo
ms: Millisecond
MRI/cMRI: Cranial Magnetic Resonance Imaging
OF: Officer Military Rank in official NATO ranking
OR: Other (non-officer) Military Rank in official NATO ranking: OR 1–4 = Enlisted ranks, OR 4–9 = Noncomissioned officers
Para: Parasubiculum
Pre: Presubiculum
PTSD: Post-Traumatic Stress Disorder
SD: Standard Deviation
SUB: Subiculum
T: Tesla
TE: Time to Echo
TR: Repetition Time
