# Supplementary material for: Cross-sectional field study comparing hippocampal subfields in patients with post-traumatic stress disorder, major depressive disorder, post-traumatic stress disorder with comorbid major depressive disorder, and adjustment disorder using routine clinical data
Source: Front Psychol. 2023 Jun 13;14:1123079. doi: 10.3389/fpsyg.2023.1123079 (PMC10299169; doi:10.3389/fpsyg.2023.1123079)
Supplement: Supplementary file 1 [file Table_1.docx]

Supplementary **Table 1.** *Results of ANCOVA parameters with and without bootstrapping.*

|  |  | CA1 | | | | | | | | |  | CA2/3 | | | | | | | | |  | DG | | | | | | | | |
| --- | --- | --- | --- | --- | --- | --- | --- | --- | --- | --- | --- | --- | --- | --- | --- | --- | --- | --- | --- | --- | --- | --- | --- | --- | --- | --- | --- | --- | --- | --- |
| Model |  | *b* |  | 95% CI | | |  | *t* |  | *p* |  | *b* |  | 95% CI | | |  | *t* |  | *p* |  | *b* |  | 95% CI | | |  | *t* |  | *p* |
| ANCOVA  with Bootstrapping | | |  |  |  |  |  |  |  |  |  |  |  |  |  |  |  |  |  |  |  |  |  |  |  |  |  |  |  |  |
| Intercept |  | 449.6 |  | [239.0 | ; | 653.7] |  |  | < | .001 |  | 123.5 |  | [24.3 | ; | 222.4] |  |  |  | .015 |  | 190.2 |  | [103.9 | ; | 296.7] |  |  | < | .001 |
| eTIV |  | 0.001 |  | [0.0001 | ; | 0.001] |  |  | < | .001 |  | 0.001 |  | [0.0002 | ; | 0.0001] |  |  | < | .001 |  | 0.0002 |  | [0.0002 | ; | 0.0001] |  |  | < | .001 |
|  |  |  |  |  |  |  |  |  |  |  |  |  |  |  |  |  |  |  |  |  |  |  |  |  |  |  |  |  |  |  |
| Patient group  reference variable: AdjD | | | | |  |  |  |  |  |  |  |  |  |  |  |  |  |  |  |  |  |  |  |  |  |  |  |  |  |  |
| PTSD |  | 3.9 |  | [-55.3 | ; | 61.3] |  |  |  | .903 |  | 15.0 |  | [-5.8 | ; | 36.5] |  |  |  | .157 |  | 9.8 |  | [-11.8 | ; | 32.1] |  |  |  | .363 |
| MDD |  | 17.8 |  | [-39.1 | ; | 74.1] |  |  |  | .549 |  | 12.6 |  | [-7.7 | ; | 32.9] |  |  |  | .216 |  | 11.9 |  | [-9.8 | ; | 34.1] |  |  |  | .276 |
| PTSD+MDD |  | -9.2 |  | [-69.7 | ; | 47.5] |  |  |  | .746 |  | 21.4 |  | [-1.8 | ; | 44.8] |  |  |  | .086 |  | 19.8 |  | [-5.1 | ; | 44.0] |  |  |  | .127 |
|  |  |  |  |  |  |  |  |  |  |  |  |  |  |  |  |  |  |  |  |  |  |  |  |  |  |  |  |  |  |  |
| ANCOVA  without Bootstrapping | | | | |  |  |  |  |  |  |  |  |  |  |  |  |  |  |  |  |  |  |  |  |  |  |  |  |  |  |
| Intercept |  | 449.6 |  | [220.6 | ; | 678.7] |  | 3.87 | < | .001 |  | 123.5 |  | [31.8 | ; | 215.3] |  | 2.6 |  | .009 |  | 190.2 |  | [100.1 | ; | 280.2] |  | 4.1 | < | .001 |
| eTIV |  | 0.001 |  | [0.00004 | ; | 0.001] |  | 8.8 | < | .001 |  | 0.0002 |  | [0.0002 | ; | 0.0001] |  | 7.7 | < | .001 |  | 0.0002 |  | [0.0002 | ; | 0.0001] |  | 9.9 | < | .001 |
| Patient group  reference variable: AdjD | | | | | |  |  |  |  |  |  |  |  |  |  |  |  |  |  |  |  |  |  |  |  |  |  |  |  |  |
| PTSD |  | 3.9 |  | [-55.6 | ; | 63.6] |  | 0.1 |  | .896 |  | 15.0 |  | [-8.8 | ; | 38.9] |  | 1.2 |  | .216 |  | 9.8 |  | [-13.5 | ; | 33.3] |  | 0.8 |  | .406 |
| MDD |  | 17.8 |  | [-39.1 | ; | 74.8] |  | 0.6 |  | .537 |  | 12.6 |  | [-10.1 | ; | 35.4] |  | 1.0 |  | .276 |  | 11.9 |  | [-10.4 | ; | 34.3] |  | 1.0 |  | .293 |
| PTSD+MDD |  | -9.2 |  | [-72.2 | ; | 53.7] |  | -0.2 |  | .773 |  | 21.4 |  | [-3.8 | ; | 46.6] |  | 1.6 |  | .096 |  | 19.8 |  | [-4.9 | ; | 44.6] |  | 1.5 |  | .116 |

*Note.* Model parameters are based on 1000 bootstrap samples. Bias-corrected confidence intervals have been applied
