# Supplementary material for: Cross-sectional field study comparing hippocampal subfields in patients with post-traumatic stress disorder, major depressive disorder, post-traumatic stress disorder with comorbid major depressive disorder, and adjustment disorder using routine clinical data
Source: Front Psychol. 2023 Jun 13;14:1123079. doi: 10.3389/fpsyg.2023.1123079 (PMC10299169; doi:10.3389/fpsyg.2023.1123079)
Supplement: Supplementary file 2 [file Table_2.docx]

**Supplementary Table 2.** *Descriptive statistics on eTIV-corrected subfields of the hippocampus. (Voxel measure).*

|  |  | MDD  (n = 70) | | |  | PTSD  (n = 50) | | |  | PTSD+MDD  (n = 38) | | |  | AdjD  (n = 27) | | |
| --- | --- | --- | --- | --- | --- | --- | --- | --- | --- | --- | --- | --- | --- | --- | --- | --- |
| Variable |  | M | 95% CI | |  | M | 95% CI | |  | M | 95% CI | |  | M | 95% CI | |
| Left |  |  |  | |  |  |  | |  |  |  | |  |  |  | |
| Para |  | 75.0 | [72.3, | 77.7] |  | 73.3 | [70.2, | 76.4] |  | 77.2 | [73.5, | 81.0] |  | 75.0 | [70.7, | 79.2] |
| Pre |  | 335.5 | [326.3, | 344.6] |  | 336.8 | [326.3, | 347.4] |  | 340.9 | [328.1, | 353.6] |  | 331.6 | [317.2, | 346.0] |
| Subiculum |  | 464.0 | [452.7, | 475.2] |  | 469.2 | [456.3, | 482.1] |  | 474.7 | [459.0, | 490.3] |  | 463.4 | [445.7, | 481.1] |
| CA1 |  | 722.2 | [705.7, | 738.6] |  | 714.6 | [695.7, | 733.4] |  | 705.3 | [682.4, | 728.3] |  | 709.1 | [683.2, | 734.9] |
| CA2/3 |  | 233.1 | [227.5, | 238.7] |  | 233.7 | [227.3, | 240.1] |  | 233.7 | [225.9, | 241.5] |  | 225.6 | [216.8, | 234.4] |
| CA4 |  | 270.4 | [265.3, | 275.5] |  | 269.1 | [263.3, | 274.9] |  | 272.6 | [265.6, | 279.7] |  | 266.3 | [258.3, | 274.2] |
| GC/DG |  | 318.2 | [312.5, | 324.0] |  | 316.3 | [309.7, | 322.8] |  | 319.9 | [311.9, | 327.8] |  | 310.7 | [301.7, | 319.7] |
| ML |  | 615.4 | [604.0, | 626.9] |  | 616.2 | [603.1, | 629.3] |  | 615.6 | [599.7, | 631.5] |  | 607.7 | [589.8, | 625.7] |
| HATA |  | 70.9 | [69.1, | 72.7] |  | 69.9 | [67.9, | 72.0] |  | 70.0 | [67.5, | 72.5] |  | 69.2 | [66.4, | 72.1] |
| Fimbria |  | 98.4 | [94.0, | 102.7] |  | 98.4 | [93.4, | 103.4] |  | 100.5 | [94.4, | 106.5] |  | 91.9 | [85.1, | 98.8] |
| HT |  | 560.2 | [545.5, | 574.9] |  | 565.8 | [548.9, | 582.6] |  | 568.6 | [548.1, | 589.1] |  | 574.6 | [551.5, | 597.7] |
| HF |  | 147.5 | [142.1, | 152.89 |  | 151.2 | [145.1, | 157.4] |  | 147.1 | [139.7, | 154.6] |  | 149.1 | [140.7, | 157.5] |
|  |  |  |  | |  |  |  | |  |  |  | |  |  |  | |
| Right |  |  |  | |  |  |  | |  |  |  | |  |  |  | |
| Para |  | 73.9 | [71.6, | 76.2] |  | 71.7 | [69.0, | 74.3] |  | 74.4 | [71.2, | 77.6] |  | 75.3 | [71.7, | 78.9] |
| Pre |  | 321.3 | [313.6, | 329.1] |  | 318.8 | [309.9, | 327.8] |  | 318.6 | [307.8, | 329.5] |  | 314.3 | [302.0, | 326.5] |
| Subiculum |  | 463.7 | [454.0, | 473.5] |  | 460.3 | [449.1, | 471.5] |  | 470.1 | [456.4, | 483.7] |  | 454.2 | [438.8, | 469.5] |
| CA1 |  | 756.2 | [739.2, | 773.2] |  | 745.2 | [725.7, | 764.7] |  | 744.8 | [721.1, | 768.5] |  | 746.1 | [719.3, | 772.9] |
| CA2/3 |  | 257.3 | [250.7, | 264.0] |  | 257.6 | [250.0, | 265.2] |  | 261.7 | [252.4, | 270.9] |  | 249.9 | [239.5, | 260.4] |
| CA4 |  | 284.2 | [278.4, | 289.9] |  | 282.7 | [276.1, | 289.4] |  | 288.3 | [280.2, | 296.3] |  | 278.3 | [269.2, | 287.4] |
| GC/DG |  | 330.7 | [324.3, | 337.2] |  | 329.1 | [321.7, | 336.6] |  | 335.1 | [326.1, | 344.1] |  | 323.9 | [313.7, | 334.1] |
| ML |  | 634.1 | [622.2, | 645.9] |  | 630.7 | [617.1, | 644.3] |  | 636.1 | [619.5, | 652.6] |  | 624.3 | [605.6, | 643.0] |
| HATA |  | 74.6 | [72.6, | 76.6] |  | 73.2 | [70.9, | 75.5] |  | 73.1 | [70.2, | 75.9] |  | 71.2 | [68.0, | 74.4] |
| Fimbria |  | 91.7 | [87.0, | 96.3] |  | 89.4 | [84.1, | 94.7] |  | 91.1 | [84.6, | 97.5] |  | 89.5 | [82.2, | 96.8] |
| HT |  | 586.5 | [571.2, | 601.8] |  | 585.9 | [568.3, | 603.4] |  | 596.7 | [575.3, | 618.0] |  | 591.0 | [566.9, | 615.1] |
| HF |  | 151.0 | [145.2, | 156.9] |  | 150.5 | [143.8, | 157.2] |  | 151.6 | [143.5, | 159.7] |  | 142.4 | [133.3, | 151.6] |
| *Note.* Para = Parasubiculum; Pre = Presubiculum; CA = Cornu Ammonis; GC/DG = Granular Cell Layer / Dentate Gyrus; ML = Molecular Layer; HATA = Hippocampus-Amygdala Transition Area; HT = Hippocampal Tail; HF = Hippocampal Fimbria; M = Mean value; CI = Confidence interval. | | | | | | | | | | | | | | | | |

**Supplementary Table 2*.*** *Continued.*

|  |  | MDD  (n = 70) | | |  | PTSD  (n = 50) | | |  | PTSD+MDD  (n = 38) | | |  | AdjD  (n = 27) | | |
| --- | --- | --- | --- | --- | --- | --- | --- | --- | --- | --- | --- | --- | --- | --- | --- | --- |
| Variable |  | M | 95% CI | |  | M | 95% CI | |  | M | 95% CI | |  | M | 95% CI | |
| Left+Right |  |  |  | |  |  |  | |  |  |  | |  |  |  | |
| Para |  | 148.9 | [144.7, | 153.2] |  | 145.0 | [140.1, | 149.9] |  | 151.6 | [145.7, | 157.6] |  | 150.3 | [143.5, | 157.0] |
| Pre |  | 656.8 | [641.5, | 672.2] |  | 655.7 | [638.1, | 673.2] |  | 659.5 | [638.2, | 680.9] |  | 645.8 | [621.8, | 669.9] |
| Subiculum |  | 927.7 | [908.2, | 947.3] |  | 929.5 | [907.1, | 951.9] |  | 944.7 | [917.5, | 971.9] |  | 917.6 | [886.9, | 948.2] |
| CA1 |  | 1478.4 | [1448.0, | 1508.8] |  | 1459.8 | [1424.9, | 1494.6] |  | 1450.1 | [1407.8, | 1492.4] |  | 1455.2 | [1407.4, | 1502.9] |
| CA2/3 |  | 490.5 | [479.8, | 501.2] |  | 491.3 | [479.0, | 503.6] |  | 495.3 | [480.4, | 510.3] |  | 475.5 | [458.7, | 492.4] |
| CA4 |  | 554.5 | [544.8, | 564.3] |  | 551.8 | [540.6, | 563.0] |  | 560.9 | [547.3, | 574.5] |  | 544.6 | [529.2, | 559.9] |
| GC/DG |  | 649.0 | [637.9, | 660.0] |  | 645.4 | [632.8, | 658.1] |  | 655.0 | [639.6, | 670.4] |  | 634.6 | [617.3, | 652.0] |
| ML |  | 1249.5 | [1228.1, | 1270.9] |  | 1246.9 | [1222.4, | 1271.3] |  | 1251.7 | [1222.0, | 1281.4] |  | 1232.0 | [1198.5, | 1265.6] |
| HATA |  | 145.5 | [142.3, | 148.8] |  | 143.1 | [139.4, | 146.8] |  | 143.1 | [138.5, | 147.6] |  | 140.4 | [135.3, | 145.6] |
| Fimbria |  | 190.0 | [181.9, | 198.1] |  | 187.8 | [178.5, | 197.1] |  | 191.5 | [180.3, | 202.8] |  | 181.4 | [168.7, | 194.1] |
| HT |  | 1146.7 | [1118.0, | 1175.4] |  | 1151.7 | [1118.8, | 1184.5] |  | 1165.3 | [1125.3, | 1205.2] |  | 1165.6 | [1120.5, | 1210.7] |
| HF |  | 298.5 | [288.5, | 308.5] |  | 301.8 | [290.3, | 313.2] |  | 298.7 | [284.8, | 312.6] |  | 291.5 | [275.9, | 307.2] |
| *Note.* Para = Parasubiculum; Pre = Presubiculum; CA = Cornu Ammonis; GC/DG = Granular Cell Layer / Dentate Gyrus; ML = Molecular Layer; HATA = Hippocampus-Amygdala Transition Area; HT = Hippocampal Tail; HF = Hippocampal Fimbria; M = Mean value; CI = Confidence interval. | | | | | | | | | | | | | | | | |
