# Supplementary material for: Cross-sectional field study comparing hippocampal subfields in patients with post-traumatic stress disorder, major depressive disorder, post-traumatic stress disorder with comorbid major depressive disorder, and adjustment disorder using routine clinical data
Source: Front Psychol. 2023 Jun 13;14:1123079. doi: 10.3389/fpsyg.2023.1123079 (PMC10299169; doi:10.3389/fpsyg.2023.1123079)
Supplement: Supplementary file 3 [file Table_3.docx]

**Supplementary Table 3.** *Results of ANCOVA with and without outliers.*

|  |  | Test statistics | | | | | | |
| --- | --- | --- | --- | --- | --- | --- | --- | --- |
| Variable |  | F | (df) |  | *p* | |  | η_p_² |
| CA1 with outliers |  |  |  |  |  | |  |  |
| eTIV |  | 77.6 | (1, 180) |  | < | .001 |  | .301 |
| patient group |  | 0.39 | (3, 180) |  |  | .301 |  | .006 |
| CA1 without outliers |  |  |  |  |  |  |  |  |
| eTIV |  | 67.9 | (1, 178) |  | < | .001 |  | .276 |
| patient group |  | 0.44 | (3, 178) |  |  | .725 |  | .007 |
| CA2/3 with outliers |  |  |  |  |  | |  |  |
| eTIV |  | 59.7 | (1, 180) |  | < | .001 |  | .249 |
| patient group |  | 0.96 | (3, 180) |  |  | .412 |  | .016 |
| CA2/3 without outliers |  |  |  |  |  |  |  |  |
| eTIV |  | 40.4 | (1, 176) |  | < | .001 |  | .187 |
| patient group |  | 1.06 | (3, 176) |  |  | .364 |  | .018 |
| DG with outliers |  |  |  |  |  | |  |  |
| eTIV |  | 98.6 | (1, 180) |  | < | .001 |  | .354 |
| patient group |  | 0.85 | (3, 180) |  |  | .467 |  | .014 |
| DG without outliers |  |  |  |  |  |  |  |  |
| eTIV |  | 86.8 | (1, 179) |  | < | .001 |  | .327 |
| patient group |  | 0.86 | (3, 179) |  |  | .463 |  | .014 |
